# *Mycobacterium chelonae-abscessus* Complex Associated with Sinopulmonary Disease, Northeastern USA

**DOI:** 10.3201/eid1709.101667

**Published:** 2011-09

**Authors:** Keith E. Simmon, Barbara A. Brown-Elliott, Perry G. Ridge, Jacob D. Durtschi, Linda Bridge Mann, E. Susan Slechta, Arnold G. Steigerwalt, Benjamin D. Moser, Anne M. Whitney, June M. Brown, Karl V. Voelkerding, Karin L. McGowan, Anne F. Reilly, Thomas J. Kirn, W. Ray Butler, Paul H. Edelstein, Richard J. Wallace, Cathy A. Petti

**Affiliations:** Author affiliations: Associated Regional and University Pathologists Institute for Clinical and Experimental Pathology, Salt Lake City, Utah, USA (K.E. Simmon, P.G. Ridge, J.D. Durtschi, E.S. Slechta, K.V. Voelkerding, C.A. Petti);; University of Texas Health Science Center, Tyler, Texas, USA (B.A. Brown-Elliott, L.B. Mann, R.J. Wallace, Jr.);; Centers for Disease Control and Prevention, Atlanta, Georgia, USA (A.G. Steigerwalt, B.D. Moser, A.M. Whitney, J.M. Brown, W.R. Butler);; University of Utah School of Medicine, Salt Lake City, Utah, USA (K.V. Voelkerding, C.A. Petti);; University of Pennsylvania School of Medicine at Children’s Hospital of Philadelphia, Philadelphia, Pennsylvania, USA (K.F. McGowan, A.F. Reilly);; Robert Wood Johnson Medical School, New Brunswick, New Jersey, USA (T.J. Kirn);; University of Pennsylvania Medical Center, Philadelphia (P.H. Edelstein)

**Keywords:** bacteria, rapid-growing mycobacteria, multilocus sequencing, Mycobacterium chelonae-abscessus complex, Mycobacterium franklinii, sinopulmonary disease, tuberculosis and other mycobacteria, United States, CME, research, *Suggested citation for this article*: Simmon KE, Brown-Elliott BA, Ridge PG, Durtschi JD, Mann LB, Slechta ES, et al. *Mycobacterium chelonae-abscessus* complex associated with sinopulmonary disease, northeastern USA. Emerg Infect Dis [serial on the Internet]. 2011 Sep [*date cited*]. http://dx.doi.org/10.3201/eid1709.101667

## MEDSCAPE CME

Medscape, LLC is pleased to provide online continuing medical education (CME) for this journal article, allowing clinicians the opportunity to earn CME credit.

This activity has been planned and implemented in accordance with the Essential Areas and policies of the Accreditation Council for Continuing Medical Education through the joint sponsorship of Medscape, LLC and Emerging Infectious Diseases. Medscape, LLC is accredited by the ACCME to provide continuing medical education for physicians.

Medscape, LLC designates this Journal-based CME activity for a maximum of 1 *AMA PRA Category 1 Credit(s)*^TM^. Physicians should claim only the credit commensurate with the extent of their participation in the activity.

All other clinicians completing this activity will be issued a certificate of participation. To participate in this journal CME activity: (1) review the learning objectives and author disclosures; (2) study the education content; (3) take the post-test with a 70% minimum passing score and complete the evaluation at www.medscape.org/journal/eid; (4) view/print certificate.

**Release date: August 22, 2011; Expiration date: August 22, 2012**

## Learning Objectives

Upon completion of this activity, participants will be able to:

- Analyze the *M. chelonae-abscessus* complex
- Distinguish the molecular identity of “*M. franklinii*”
- Identify the most common clinical source of “*M. franklinii*”
- Evaluate the antimicrobial susceptibility of “*M. franklinii*”

## MEDSCAPE CME EDITOR

**Beverly Merritt, **Technical Writer/Editor, *Emerging Infectious Diseases. Disclosure: Beverly Merritt has disclosed no relevant financial relationships*.

## MEDSCAPE CME AUTHOR

**Charles P. Vega, MD**, Associate Professor; Residency Director, Department of Family Medicine, University of California, Irvine. *Disclosure: Charles P. Vega, MD, has disclosed no relevant financial relationships.*

## AUTHORS

*Disclosures:* ***Barbara A. Brown-Elliott, MS; Perry G. Ridge; Jacob D. Durtschi, BS; Linda Bridge Mann, BS; E. Susan Slechta; Arnold G. Steigerwalt, BS; Benjamin D. Moser; Anne M. Whitney, PhD; June M. Brown, BS; Karl V. Voelkerding, MD; Karin L. McGowan, PhD; Anne F. Reilly, MD, MPH; W. Ray Butler, MS; Paul H. Edelstein, MD;*** *and* ***Richard J. Wallace Jr., MD*** *, have disclosed no relevant financial relationships.* ***Keith E. Simmon, BS,*** *has disclosed the following relevant financial relationships: employed by: Isentio US/Software company (non-FDA).* ***Thomas J. Kirn, MD,*** *has disclosed the following relevant financial relationships: served as an advisor or consultant for Merck Sharpe and Dohme.* ***Cathy A. Petti, MD,*** *has disclosed the following relevant financial relationships: was an employee for Novartis Diagnostics from 2009–2010.*
