# Supplementary material for: Mycobacterium chelonae-abscessus Complex Associated with Sinopulmonary Disease, Northeastern USA
Source: Emerg Infect Dis. 2011 Sep;17(9):1692–700. doi: 10.3201/eid1709.101667 (PMC3322061; doi:10.3201/eid1709.101667)
Supplement: Technical Appendix — Study isolates, gene designations, similarity tables, DNA comparison, and case history information pertaining to the article. [file 10-1667-Techapp_7p.pdf]

Article DOI: http://dx.doi.org/10.3201/eid1709.101667

# *Mycobacterium chelonae-abscessus* Complex Associated with Sinopulmonary Disease, Northeastern USA

## Technical Appendix

Technical Appendix Table 1. Study isolates and sequevar group for the 16S rRNA, *rpoB, sodA, hsp65* genes and ITS region of *the Mycobacterium chelonae-abscessus* complex *

| Isolate no. | 16S rRNA sequevar group | *rpoB* sequavar group | *sodA* sequevar group | *hsp65* sequevar group | *ITS* sequevar group |
|---|---|---|---|---|---|
| *M. abscessus* isolates | | | | | |
| A001 | ABS[A] | ABS[C] | ABS[A] | ABS[A] | ABS[C] |
| A003 | ABS[A] | ABS[A] | ABS[A] | ABS[A] | ABS[C] |
| A004 | ABS[A] | ABS[C] | ABS[B] | ABS[B] | ABS[D] |
| A005 | ABS[A] | ABS[C] | ABS[B] | ABS[B] | ABS[D] |
| A006 | ABS[A] | ABS[C] | ABS[B] | ABS[B] | ABS[D] |
| A007 | ABS[A] | ABS[C] | ABS[B] | ABS[B] | ABS[D] |
| A010 | ABS[A] | ABS[C] | ABS[B] | ABS[B] | ABS[D] |
| A011 | ABS[A] | ABS[C] | ABS[B] | ABS[B] | ABS[D] |
| A014 | ABS[A] | ABS[C] | ABS[B] | ABS[B] | ABS[D] |
| A015 | ABS[A] | ABS[C] | ABS[B] | ABS[B] | ABS[D] |
| A017 | ABS[A] | ABS[C] | ABS[B] | ABS[B] | ABS[D] |
| A020 | ABS[A] | ABS[C] | ABS[B] | ABS[B] | ABS[D] |
| A021 | ABS[B] | ABS[C] | ABS[B] | ABS[B] | ABS[D] |
| A023 | ABS[A] | ABS[C] | ABS[B] | ABS[B] | ABS[D] |
| A024 | ABS[A] | ABS[C] | ABS[B] | ABS[B] | ABS[D] |
| A029 | ABS[A] | ABS[C] | ABS[A] | ABS[A] | ABS[C] |
| A030 | ABS[A] | ABS[C] | ABS[B] | ABS[B] | ABS[D] |
| A032 | ABS[A] | ABS[C] | ABS[B] | ABS[B] | ABS[D] |
| A033 | ABS[A] | ABS[C] | ABS[B] | ABS[B] | ABS[D] |
| A034 | ABS[A] | ABS[C] | ABS[B] | ABS[B] | ABS[D] |
| A035 | ABS[A] | ABS[C] | ABS[B] | ABS[B] | ABS[D] |
| A036 | ABS[A] | ABS[C] | ABS[B] | ABS[B] | ABS[D] |
| A039 | ABS[A] | ABS[C] | ABS[B] | ABS[B] | ABS[D] |
| A041 | ABS[A] | ABS[C] | ABS[B] | ABS[B] | ABS[D] |
| A042 | ABS[A] | ABS[C] | ABS[B] | ABS[B] | ABS[D] |
| A043 | ABS[A] | ABS[C] | ABS[A] | ABS[A] | ABS[C] |
| A044 | ABS[A] | ABS[C] | ABS[B] | ABS[B] | ABS[D] |
| A046 | ABS[A] | ABS[C] | ABS[B] | ABS[B] | ABS[D] |
| A047 | ABS[A] | ABS[C] | ABS[B] | ABS[B] | ABS[D] |
| A048 | ABS[A] | ABS[C] | ABS[B] | ABS[B] | ABS[D] |
| A049 | ABS[A] | ABS[C] | ABS[B] | ABS[B] | ABS[D] |
| A050 | ABS[A] | ABS[C] | ABS[B] | ABS[B] | ABS[D] |
| A051 | ABS[A] | ABS[C] | ABS[B] | ABS[B] | ABS[D] |
| A052 | ABS[A] | ABS[C] | ABS[B] | ABS[B] | ABS[D] |
| A053 | ABS[A] | ABS[D] | ABS[C] | ABS[B] | ABS[D] |
| A054 | ABS[A] | ABS[B] | ABS[B] | ABS[B] | ABS[A] |
| A056 | ABS[A] | ABS[C] | ABS[B] | ABS[B] | ABS[D] |
| A057 | ABS[A] | ABS[C] | ABS[A] | ABS[A] | ABS[C] |
| A058 | ABS[A] | ABS[C] | ABS[A] | ABS[A] | ABS[C] |
| A059 | ABS[A] | ABS[C] | ABS[B] | ABS[B] | ABS[D] |
| A060 | ABS[A] | ABS[C] | ABS[A] | ABS[A] | ABS[C] |
| A061 | ABS[A] | ABS[C] | ABS[B] | ABS[B] | ABS[D] |
| A063 | ABS[A] | ABS[C] | ABS[B] | ABS[B] | ABS[B] |
| A064 | ABS[A] | ABS[C] | ABS[C] | ABS[B] | ABS[D] |

| | | | | | |
|---|---|---|---|---|---|
| A065 | ABS[A] | ABS[C] | ABS[C] | ABS[B] | ABS[D] |
| A066 | ABS[A] | ABS[C] | ABS[C] | ABS[B] | ABS[D] |
| A067 | ABS[A] | ABS[D] | ABS[C] | ABS[B] | ABS[D] |
| A068 | ABS[A] | ABS[D] | ABS[C] | ABS[B] | ABS[D] |
| A069 | ABS[A] | ABS[C] | ABS[B] | ABS[B] | ABS[D] |
| A070 | ABS[A] | ABS[D] | ABS[C] | ABS[B] | ABS[D] |
| A071 | ABS[A] | ABS[D] | ABS[C] | ABS[B] | ABS[D] |
| A072 | ABS[A] | ABS[D] | ABS[C] | ABS[B] | ABS[D] |
| A073 | ABS[A] | ABS[C] | ABS[D] | ABS[C] | ABS[D] |
| A074 | ABS[A] | ABS[D] | ABS[C] | ABS[B] | ABS[A] |
| A075 | ABS[A] | ABS[D] | ABS[C] | ABS[B] | ABS[A] |
| A076 | ABS[A] | ABS[D] | ABS[C] | ABS[B] | ABS[A] |
| A077 | ABS[A] | ABS[D] | ABS[C] | ABS[B] | ABS[D] |
| A078 | ABS[A] | ABS[D] | ABS[C] | ABS[B] | ABS[A] |
| A079 | ABS[A] | ABS[C] | ABS[C] | ABS[B] | ABS[D] |
| A080 | ABS[A] | ABS[C] | ABS[B] | ABS[B] | ABS[D] |
| A081 | ABS[B] | ABS[D] | ABS[C] | ABS[B] | ABS[D] |
| A082 | ABS[B] | ABS[D] | ABS[C] | ABS[B] | ABS[D] |
| A083 | ABS[B] | ABS[D] | ABS[C] | ABS[B] | ABS[D] |
| *M. abscessus* CIP 104536$^T$ | ABS[A] | ABS[C] | ABS[B] | ABS[B] | ABS[D] |
| *M. bolletii* isolates | | | | | |
| *M. bolletii* CIP 108541$^T$ | ABS[A] | BOL | BOL | BOL | ABS[D] |
| *M. chelonae* isolates | | | | | |
| C001 | CHE[A] | CHE[G] | CHE[E] | CHE[C] | CHE[G] |
| C002 | CHE[A] | CHE[A] | CHE[B] | CHE[A] | CHE[E] |
| C003 | CHE[A] | CHE[A] | CHE[A] | CHE[A] | CHE[A] |
| C004 | CHE[A] | CHE[B] | CHE[A] | CHE[A] | CHE[F] |
| C005 | CHE[A] | CHE[E] | CHE[C] | CHE[A] | CHE[H] |
| C006 | CHE[A] | CHE[A] | CHE[B] | CHE[A] | CHE[E] |
| C009 | CHE[A] | CHE[G] | CHE[E] | CHE[C] | CHE[G] |
| C010 | CHE[A] | CHE[A] | CHE[A] | CHE[A] | CHE[A] |
| C011 | CHE[A] | CHE[A] | CHE[A] | CHE[A] | CHE[A] |
| C012 | CHE[A] | CHE[A] | CHE[A] | CHE[A] | CHE[A] |
| C013 | CHE[A] | CHE[F] | CHE[D] | CHE[A] | CHE[C] |
| C014 | CHE[A] | CHE[C] | CHE[B] | CHE[A] | CHE[F] |
| C015 | CHE[A] | CHE[G] | CHE[E] | CHE[C] | CHE[G] |
| C016 | CHE[D] | CHE[H] | CHE[E] | CHE[C] | CHE[I] |
| C017 | CHE[A] | CHE[A] | CHE[A] | CHE[A] | CHE[A] |
| C018 | CHE[A] | CHE[A] | CHE[A] | CHE[A] | CHE[A] |
| C019 | CHE[A] | CHE[C] | CHE[B] | CHE[A] | CHE[F] |
| C020 | CHE[A] | CHE[E] | CHE[C] | CHE[B] | CHE[J] |
| C021 | CHE[A] | CHE[C] | CHE[B] | CHE[A] | CHE[F] |
| C022 | CHE[D] | CHE[I] | CHE[G] | CHE[C] | CHE[I] |
| C023 | CHE[A] | CHE[C] | CHE[B] | CHE[A] | CHE[F] |
| C024 | CHE[A] | CHE[A] | CHE[B] | CHE[A] | CHE[E] |
| C025 | CHE[B] | CHE[C] | CHE[B] | CHE[A] | CHE[F] |
| C026 | CHE[A] | CHE[H] | CHE[E] | CHE[D] | CHE[D] |
| C027 | CHE[A] | CHE[E] | CHE[C] | CHE[A] | CHE[H] |
| C029 | CHE[A] | CHE[G] | CHE[C] | CHE[E] | CHE[G] |
| C031 | CHE[A] | CHE[A] | CHE[B] | CHE[A] | CHE[E] |
| C032 | CHE[C] | CHE[A] | CHE[A] | CHE[A] | CHE[A] |
| C033 | CHE[A] | CHE[G] | CHE[C] | CHE[E] | CHE[G] |
| C034 | CHE[B] | CHE[C] | CHE[B] | CHE[A] | CHE[F] |
| C035 | CHE[A] | CHE[C] | CHE[B] | CHE[A] | CHE[F] |
| C038 | CHE[A] | CHE[C] | CHE[B] | CHE[A] | CHE[F] |
| C039 | CHE[A] | CHE[A] | CHE[B] | CHE[A] | CHE[B] |
| C040 | CHE[A] | CHE[C] | CHE[B] | CHE[A] | CHE[F] |
| C041 | CHE[A] | CHE[C] | CHE[B] | CHE[A] | CHE[F] |
| C042 | CHE[A] | CHE[C] | CHE[B] | CHE[A] | CHE[F] |
| C043 | CHE[A] | CHE[A] | CHE[H] | CHE[A] | CHE[E] |
| C044 | CHE[D] | CHE[I] | CHE[G] | CHE[C] | CHE[I] |
| C045 | CHE[A] | CHE[E] | CHE[C] | CHE[A] | CHE[H] |
| C047 | CHE[A] | CHE[A] | CHE[A] | CHE[A] | CHE[A] |
| C048 | CHE[A] | CHE[C] | CHE[B] | CHE[A] | CHE[F] |
| C050 | CHE[A] | CHE[D] | CHE[C] | CHE[E] | CHE[G] |
| C051 | CHE[D] | CHE[I] | CHE[G] | CHE[C] | CHE[I] |

| | | | | | |
|---|---|---|---|---|---|
| C052 | CHE[A] | CHE[C] | CHE[B] | CHE[A] | CHE[F] |
| C053 | CHE[A] | CHE[A] | CHE[A] | CHE[A] | CHE[A] |
| C054 | CHE[A] | CHE[A] | CHE[B] | CHE[A] | CHE[E] |
| C055 | CHE[A] | CHE[E] | CHE[C] | CHE[A] | CHE[H] |
| C056 | CHE[A] | CHE[C] | CHE[B] | CHE[A] | CHE[F] |
| C057 | CHE[A] | CHE[A] | CHE[A] | CHE[A] | CHE[A] |
| C058 | CHE[A] | CHE[C] | CHE[B] | CHE[A] | CHE[F] |
| C059 | CHE[A] | CHE[A] | CHE[B] | CHE[A] | CHE[E] |
| C060 | CHE[A] | CHE[G] | CHE[F] | CHE[E] | CHE[H] |
| C061 | CHE[A] | CHE[E] | CHE[C] | CHE[A] | CHE[F] |
| C062 | CHE[A] | CHE[C] | CHE[B] | CHE[A] | CHE[H] |
| C063 | CHE[A] | CHE[C] | CHE[B] | CHE[A] | CHE[F] |
| C064 | CHE[A] | CHE[E] | CHE[C] | CHE[A] | CHE[H] |
| C065 | CHE[A] | CHE[H] | CHE[I] | CHE[E] | CHE[K] |
| C066 | CHE[A] | CHE[C] | CHE[B] | CHE[A] | CHE[F] |
| *M. chelonae* ATCC 35758[T] | CHE[E] | CHE[J] | CHE[J] | CHE[F] | CHE[L] |
| *M.immunogenum* isolates | | | | | |
| *M. immunogenum* CIP 106684[T] | IMM | IMM | IMM | IMM | IMM |
| *M. massiliense* isolates | | | | | |
| M001 | MAS[B] | MAS[A] | ABS[A] | ABS[A] | ABS[C] |
| M002 | MAS[B] | MAS[A] | ABS[A] | ABS[A] | ABS[C] |
| M003 | ABS[A] | MAS[B] | ABS[A] | ABS[A] | ABS[C] |
| M004 | ABS[A] | MAS[B] | ABS[A] | ABS[A] | ABS[C] |
| M005 | MAS[B] | MAS[A] | ABS[A] | ABS[A] | ABS[C] |
| *M. massiliense* CCUG 48898[T] | ABS[A] | MAS[C] | ABS[A] | ABS[A] | ABS[C] |
| Novel species | | | | | |
| CV002 | CHE[E] | CV[B] | CV[B] | CV[B] | CV[B] |
| CV004 | CHE[E] | CV [A] | CV [A] | CV [A] | CV [A] |
| CV005 | CHE[E] | CV[B] | CV[B] | CV[B] | CV[B] |
| CV006 | CHE[E] | CV [A] | CV [A] | CV [A] | CV [A] |
| CV007 | CHE[E] | CV[B] | CV[B] | CV[B] | CV[B] |
| CV008 | CHE[E] | CV[B] | CV[B] | CV[B] | CV[B] |
| CV010 | CHE[E] | CV[B] | CV[B] | CV[B] | CV[B] |
| CV012 | CHE[E] | CV[B] | CV[B] | CV[B] | CV[B] |
| CV013 | CHE[E] | CV [A] | CV [A] | CV [A] | CV [A] |
| CV014 | CHE[E] | CV [A] | CV [A] | CV [A] | CV [A] |
| CV015 | CHE[E] | CV [A] | CV [A] | CV [A] | CV [A] |
| CV021 | NA | CV[B] | NA | NA | NA |
| CV022 | NA | CV[D] | NA | NA | NA |
| CV023 | NA | CV[D] | NA | NA | NA |
| CV024 | NA | CV [A] | NA | NA | NA |
| CV025 | NA | CV[C] | NA | NA | NA |
| CV026 | NA | CV[D] | NA | NA | NA |
| CV027 | NA | CV [A] | NA | NA | N/ |
| CV028 | NA | CV[B] | NA | NA | NA |
| CV030 | NA | CV[B] | NA | NA | NA |
| CV031 | NA | CV[C] | NA | NA | NA |
| CV032 | NA | CV[C] | NA | NA | NA |
| CV033 | NA | CV[B] | NA | NA | NA |
| CV034 | NA | CV[B] | NA | NA | NA |
| CV035 | NA | CV[B] | NA | NA | NA |
| CV036 | NA | CV[A] | NA | NA | NA |

*ITS, internal transcribed spacer; CIP, Collection of Institut Pasteur; NA, not applicable.

Technical Appendix Table 2. Gene designations and corresponding GenBank accession numbers of study isolates and reference sequences from *Mycobacterium abscessus**

| Gene name | Alignment gene length, bp | *M. abscessus* ORF designation | *M. abscessus* (NC_010397) Protein ID# | GenBank accession nos. for study isolates |
|---|---|---|---|---|
| Translation initiation factor IF-3 (*infC*) | 531 | MAB_2321 | YP_001703056 | HQ661899– HQ661904 |
| S-adenosyl-methyltransferase (*mraW*) | 1014 | MAB_1998 | YP_001702734 | HQ661905– HQ661910 |
| Phosphatidate cytidylyltransferase (*cdsA*) | 879 | MAB_3186c | YP_001703917 | HQ661911– HQ661916 |
| Phosphoribosylaminoimidazole carboxylase (*purE*) | 501 | MAB_3619c | YP_001704347 | HQ661917– HQ661922 |
| Preprotein translocase subunit (*secY*) | 1260 | MAB_3784c | YP_001704513 | HQ661923– HQ661928 |
| (HSP-70 cofactor (*grpE*) | 753 | MAB_4272c | YP_001704999 | HQ661929– HQ661934 |
| Recombination protein (*recR*) | 609 | MAB_0320 | YP_001701073 | HQ661935–HQ661940 |
| Ribonuclease P protein component (*rnpA*) | 384 | MAB_4954c | YP_001705676 | HQ661941–HQ661946 |
| Elongation factor Ts (*tsf*) | 834 | MAB_3195c | YP_001703926 | HQ661947–HQ661952 |
| Hypothetical protein Lactamase_B | 1692 | MAB_3083c | YP_001703815 | HQ661953–HQ661958 |
| 50S ribosomal protein L7/L12 (*rplL*) | 390 | MAB_3876c | YP_001704604 | HQ661959–HQ661964 |
| 50S ribosomal protein L13 (*rplM*) | 444 | MAB_3752c | YP_001704480 | HQ661965–HQ661970 |
| Co-chaperonin GroES (*groE*S) | 300 | MAB_3732c | YP_001704460 | HQ661971–HQ661976 |
| 30S ribosomal protein S1 *(rpsA*) | 1446 | MAB_2296 | YP_001703031 | HQ661977–HQ661982 |
| Phosphoribosylformylglycinamidine synthase II (*purL*) | 2286 | MAB_0707 | YP_001701457 | HQ661983–HQ661988 |
| Putative Mrp homolog protein | 1137 | MAB_1366c | YP_001702106 | HQ661989–HQ661994 |
| Putative metalloprotease | 513 | MAB_1669 | YP_001702408 | HQ661995–HQ662000 |
| 50S ribosomal protein L20 (*rplT*) | 390 | MAB_2323 | YP_001703058 | HQ662001–HQ662006 |
| ABC transporter ATP-binding protein | 720 | MAB_2747c | YP_001703480 | HQ662007–HQ662012 |
| Guanylate kinase | 501 | MAB_2823c | YP_001703556 | HQ662013–HQ662018 |
| Elongation factor P (*ef*P) | 564 | MAB_2837c | YP_001703570 | HQ662019–HQ662024 |
| Deoxyuridine 5'-triphosphate nucleotidohydrolase (*dut*) | 453 | MAB_3003c | YP_001703735 | HQ662025–HQ662030 |
| 30s ribosomal protein S2 (*rpsB*) | 849 | MAB_3196c | YP_001703927 | HQ662031–HQ662036 |
| Aspartyl/glutamyl-tRNA amidotransferase, B subunit (*gatB*) | 1482 | MAB_3334c | YP_001704064 | HQ662037–HQ662042 |
| 30s ribosomal protein S9 (*rpsI*) | 534 | MAB_3751c | YP_001704479 | HQ662043–HQ662048 |
| 50S ribosomal protein L6 (*rplF*) | 540 | MAB_3797c | YP_001704526 | HQ662049–HQ662054 |
| Transcription antitermination protein NusG (*nusG*) | 804 | MAB_3894c | YP_001704622 | HQ662055–HQ662060 |
| Adenylosuccinate synthetase (*purA*) | 1296 | MAB_4249c | YP_001704976 | HQ662061–HQ662066 |
| Cell division protein FtsZ (*ftsZ*) | 1164 | MAB_2009 | YP_001702745 | HQ662067–HQ662072 |
| Recombinase A (*recA*) | 1041 | MAB_3060c | YP_001703792 | HQ662073–HQ662078 |
| Tyrosyl-tRNA synthetase (*tyrS*) | 1293 | MAB_2354 | YP_001703089 | HQ662079–HQ662084 |
| UDP-N-acetylglucosamine pyrophosphorylase (*glmU*) | 1452 | MAB_1148c | YP_001701890 | HQ662085–HQ662090 |
| Valyl-tRNA synthetase (*valS*) | 2643 | MAB_1603 | YP_001702342 | HQ662091–HQ662096 |
| Crossover junction endodeoxyribonuclease (*ruvC*) | 510 | MAB_2884c | YP_001703617 | HQ662097–HQ662102 |
| DNA-directed RNA polymerase subunit beta (*rpoC*) | 3960 | MAB_3868c | YP_001704596 | HQ662103–HQ662108 |
| DNA repair protein RecN (*recN*) | 1770 | MAB_2361 | YP_001703096 | HQ662109–HQ662114 |
| GTP-dependent nucleic acid-binding protein (*ychF*) | 1074 | MAB_1266 | YP_001702008 | HQ662115–HQ662120 |
| GTP-binding protein Era (*era*) | 915 | MAB_1672 | YP_001702411 | HQ662121–HQ662126 |
| Hypothetical protein MAB2781c | 978 | MAB_2781c | YP_001703514 | HQ662127–HQ662132 |
| Nicotinate-nucleotide adenylyltransferase (*nadD*) | 638 | MAB_1621 | YP_001702360 | HQ662133–HQ662138 |
| 50S ribosomal protein L10 (*rplJ*) | 531 | MAB_3877c | YP_001704605 | HQ662139–HQ662144 |
| 50S ribosomal protein L35 (*rpmI*) | 195 | MAB_2322 | YP_001703057 | HQ662145–HQ662150 |
| Putative Holliday junction resolvase | 522 | MAB_2850c | YP_001703583 | HQ662151–HQ662156 |

*ORF, open reading frame; ID, identification

Technical Appendix Table 3. Similarity table of the average (range) of percent identity between concatenated DNA sequences of 43 genes of the *Mycobacterium chelonae-abscessus* complex type strains and CV002*

| Strains | Average % identity (range) | | | | | |
|---|---|---|---|---|---|---|
| | *M. abscessus* CIP 104536$^{Ta}$ | *M. bolletii* CIP 108541$^{T}$ | *M. chelonae* ATCC 35752$^{T}$ | CV002 (*M. chelonae* variant) | *M. massiliense* CCUG 48898$^{T}$ | *M. immunogenum* CIP 106684$^{T}$ |
| *M. abscessus* CIP 104536$^{Ta}$ | – | 98.3 (94.4–99.8) | 89.1 (80.7–97.3) | 90.0 (83.5–97.3) | 98.2 (92.2–100) | 91.4 (83.9–98.0) |
| *M. bolletii* CIP 108541$^{T}$ | | – | 89.3 (80.7–98.0) | 90.1 (83.8–98.0) | 98.2 (95.1–100) | 91.5 (84.1–98.3) |
| *M. chelonae* ATCC 35752$^{T}$ | | | – | 90.0 (82.2–98.3) | 89.3 (81.0–97.7) | 89.6 (81.0–98.0) |
| CV02 | | | | – | 90.1 (83.8–97.7) | 90.5 (83.4–99.7) |
| *M. massiliense* CCUG 48898$^{T}$ | | + | | | – | 91.7 (83.9–99.2) |

*Sequences derived from GenBank accession no. NC_010397. CIP, Collection of Institut Pasteur.

Technical Appendix Table 4. Similarity table of the average (range) of percent identity between concatenated amino acid sequences of 43 genes of the *Mycobacterium chelonae-abscessus* complex type strains and CV002*

| Strains | Average % identity (range) | | | | | |
|---|---|---|---|---|---|---|
| | *M. abscessus* CIP 104536$^{T}$ | *M. bolletii* CIP 108541$^{T}$ | *M. chelonae* ATCC 35752$^{T}$ | CV002 (*M. chelonae* variant) | *M. massiliense* CCUG 48898$^{T}$ | *M. immunogenum* CIP 106684$^{T}$ |
| *M. abscessus* CIP 104536$^{T}$ | – | 99.6 (97.6–100) | 95.6 (83.7–100) | 95.9 (87.3–100) | 99.5 (97.8-–100) | 97.2 (88.3–100) |
| *M. bolletii* CIP 108541$^{T}$ | | – | 95.7 (84.5–100) | 95.9 (88.0–100) | 99.5 (98.2–100) | 97.2 (88.3–100) |
| *M. chelonae* ATCC 35752$^{T}$ | | | – | 95.8 (84.1–100) | 95.6 (84.5–100) | 95.9 (84.1–100) |
| CV002 | | | | – | 95.9 (88.0–100) | 96.1 (86.5–100) |
| *M. massiliense* CCUG 48898$^{T}$ | | | | | – | 97.2 (88.3–100) |

*CIP, Collection of Institut Pasteur.

Technical Appendix Table 5. Comparison of DNA-DNA hybridization results of patient isolates and phylogenetically related type strains of *the Mycobacterium chelonae-abscessus* complex*

| Unlabeled DNA | % similarity to radiolabeled CV002 | | % similarity to radiolabeled CV005 | |
|---|---|---|---|---|
| | RBR at 70°C | % Divergence | RBR at 70°C | % Divergence |
| CV002 | 100 | 0.0 | 100 | 0.5 |
| CV004 | 80 | 1.5 | | |
| CV005 | 66 | 0.0 | 100 | 0.0 |
| CV006 | 96 | 2.0 | | |
| CV015 | 94 | 2.0 | | |
| *M. abscessus* CIP 104536$^{T}$ | 66 | 6.0 | | |
| *M. bolletii* CIP 108541$^{T}$ | 15 | 6.0 | | |
| *M. chelonae* ATCC 35752$^{T}$ | 42 | 8.0 | | |
| *M. immunogenum* CIP 106684$^{T}$ | 65 | 5.0 | | |
| *M. massiliense* CCUG 48898$^{T}$ | 69 | 6.5 | | |
| *M. salmoniphilum* ATCC 13758$^{T}$ | 41 | 6.5 | | |

*RBR, relative binding ratio (see text for full definition); CIP, Collection of Institut Pasteur.

Technical Appendix Table 6. Case histories of patients with CV recovered during study*

| Isolate no. | Year isolated | Patient age, y/sex | State | Source | Underlying condition | Symptoms | Treatment | Outcome |
|---|---|---|---|---|---|---|---|---|
| CV002 | 2005 | 9/M | NY | Skin lesion | Unknown | Unknown | Unknown | Unknown |
| CV004 | 2007 | 5/M | PA | Central line port | Medulloblastoma, chemotherapy | Erythema and purulent discharge at central line site | Levofloxacin, cefoxitin, clarithromycin | Infection resolved |
| CV005 | 2007 | 2/F | PA | Bronchial wash | Esotropia, vocal cord paralysis, and gastro-duodenal fistula | Cough | Unknown | Infection resolved |
| CV006 | 2007 | 10/F | PA | Liver biopsy | Relapsed acute lymphoblastoma leukemia | Biopsy-confirmed granulomatous liver lesions | Clindamycin, ceftazidime | Infection resolved |
| CV007 | 2007 | 41/F | PA | Sputum | Primary ciliary dyskinesia, bronchiectasis and prior *Mycobacterium avium-intracellulare* and *M. abscessus* infection | Dyspnea and increased sputum for several months | Intravenous cefepime for 3 weeks | Symptoms improved |
| CV008 | 2007 | 64/F | PA | Sputum | Prednisone therapy, bronchiectasis, multiple episodes of pneumonia and autoimmune hepatitis | Dyspnea and increased sputum production for 3 weeks | 10-d course of fluoroquinolone | Respiratory function subjectively and radiographically improved |
| CV010 | 2007 | 72/F | PA | Sputum | Unknown | Unknown | Unknown | Unknown |
| CV012 | 2007 | 63/F | PA | Maxillary sinus | Unknown | Unknown | Unknown | Unknown |
| CV013 | 2007 | 45/F | PA | Sputum | Cystic fibrosis and prior *Mycobacterium avuim* infection | Worsening cough, sputum production and dyspnea. Chest CT scan showed bilateral bronchiectasis and tree in bud opacities | At time of presentation, receiving azithromycin, ethambutol and rifampin for *M. avium*; continued on same regimen for several months | Symptoms subjectively improved and follow up cultures were repeatedly negative for *M. avium* and rapidly growing mycobacteria |
| CV014 | 2007 | 74/F | PA | Sputum | Unknown | Three isolates recovered over 3- year period | Unknown | Unknown |
| CV015 | 2008 | 10/M | PA | Sputum | Eczema | Fever, sore throat, stomatitis, and drooling. HSV-1 PCR sputum/saliva positive | Acyclovir | Lost to follow-up |
| CV021 | 2008 | 76/M | NJ | Bronchial wash | Unknown | Unknown | Unknown | Unknown |
| CV022 | 2008 | 58/F | MN | Sputum | Unknown | Unknown | Unknown | Unknown |
| CV023 | 2009 | 69/M | VA | Respiratory | Unknown | Unknown | Unknown | Unknown |
| CV024 | 2009 | 78/F | ME | Respiratory | Unknown | Unknown | Unknown | Unknown |
| CV025 | 2009 | 73/F | OR | Body fluid | Unknown | Unknown | Unknown | Unknown |
| CV026 | 2009 | 18/M | CO | Sputum | Unknown | Unknown | Unknown | Unknown |
| CV027 | 2009 | 18/M | PA | Sputum | Diabetes | Lung injury following smoke inhalation from house fire. Diagnosed with pulmonary mucormycosis by BAL. Weeks later developed respiratory failure and pulmonary hemorrhage | Unknown | Patient died |
| CV028 | 2009 | 38/F | ME | Sputum | Unknown | Unknown | Unknown | Unknown |
| CV030 | 2009 | 48/F | ME | Sputum | Unknown | Unknown | Unknown | Unknown |
| CV031 | 2009 | 53/F | ND | Bronchial lavage | Unknown | Unknown | Unknown | Unknown |

| | | | | | | | | |
|---|---|---|---|---|---|---|---|---|
| CV032 | 2009 | 1/M | NJ | Blood | Unknown | Unknown | Unknown | Unknown |
| CV033 | 2009 | 73/M | PA | Maxillary sinus | Unknown | Unknown | Unknown | Unknown |
| CV034 | 2009 | 44/F | PA | Maxillary sinus | Maxillary sinus surgery for chronic sinusitis | Two weeks postoperatively had routine follow-up and complained of nasal drainage. | None | Nasal drainage resolved over several weeks without antimicrobial therapy |
| CV035 | 2010 | 66/F | PA | Thigh lesion | Diabetes | Erythematous 2-cm indurated plaque to a 3.5- x 3.5-cm lesion. First skin biopsy showed granulomatous reaction. Subsequent biopsies showed abscess and adjacent leukocytoclastic vasculitis | Unknown | Unknown |
| CV036 | 2010 | 47/M | PA | Maxillary sinus | Unknown | Unknown | Unknown | Unknown |

*CV, *Mycobacterium chelonae* variant; CT, computed tomography; HSV-1, herpes simplex virus 1.
