# Supplementary material for: Mycobacterium chelonae-abscessus Complex Associated with Sinopulmonary Disease, Northeastern USA
Source: Emerg Infect Dis. 2011 Sep;17(9):1692–700. doi: 10.3201/eid1709.101667 (PMC3322061; doi:10.3201/eid1709.101667)
Supplement: Related texts

# Research article: *Mycobacterium chelonae-abscessus* Complex Associated with Sinopulmonary Disease, Northeastern USA

**DOI:** 10.3201/eid1709.101667

**Authors:** Keith E. Simmon, Barbara A. Brown-Elliott, Perry G. Ridge, Jacob D. Durtschi, Linda Bridge Mann, E. Susan Slechta, Arnold G. Steigerwalt, Benjamin D. Moser, Anne M. Whitney, June M. Brown, Karl V. Voelkerding, Karin L. McGowan, Anne F. Reilly, Thomas J. Kirn, W. Ray Butler, Paul H. Edelstein, Richard J. Wallace, Cathy A. Petti

**Affiliations:** Author affiliations: Associated Regional and University Pathologists Institute for Clinical and Experimental Pathology, Salt Lake City, Utah, USA (K.E. Simmon, P.G. Ridge, J.D. Durtschi, E.S. Slechta, K.V. Voelkerding, C.A. Petti);; University of Texas Health Science Center, Tyler, Texas, USA (B.A. Brown-Elliott, L.B. Mann, R.J. Wallace, Jr.);; Centers for Disease Control and Prevention, Atlanta, Georgia, USA (A.G. Steigerwalt, B.D. Moser, A.M. Whitney, J.M. Brown, W.R. Butler);; University of Utah School of Medicine, Salt Lake City, Utah, USA (K.V. Voelkerding, C.A. Petti);; University of Pennsylvania School of Medicine at Children’s Hospital of Philadelphia, Philadelphia, Pennsylvania, USA (K.F. McGowan, A.F. Reilly);; Robert Wood Johnson Medical School, New Brunswick, New Jersey, USA (T.J. Kirn);; University of Pennsylvania Medical Center, Philadelphia (P.H. Edelstein)

**Keywords:** *Suggested citation for this article*: Simmon KE, Brown-Elliott BA, Ridge PG, Durtschi JD, Mann LB, Slechta ES, et al. *Mycobacterium chelonae-abscessus* complex associated with sinopulmonary disease, northeastern USA. Emerg Infect Dis [serial on the Internet]. 2011 Sep [*date cited*]. http://dx.doi.org/10.3201/eid1709.101667

## Abstract

Members of the *Mycobacterium chelonae-abscessus* complex represent *Mycobacterium* species that cause invasive infections in immunocompetent and immunocompromised hosts. We report the detection of a new pathogen that had been misidentified as *M. chelonae* with an atypical antimicrobial drug susceptibility profile. The discovery prompted a multicenter investigation of 26 patients. Almost all patients were from the northeastern United States, and most had underlying sinus or pulmonary disease. Infected patients had clinical features similar to those with *M. abscessus* infections. Taxonomically, the new pathogen shared molecular identity with members of the *M. chelonae-abscessus* complex. Multilocus DNA target sequencing, DNA-DNA hybridization, and deep multilocus sequencing (43 full-length genes) support a new taxon for these microorganisms. Because most isolates originated in Pennsylvania, we propose the name *M. franklinii* sp. nov. This investigation underscores the need for accurate identification of *Mycobacterium* spp. to detect new pathogens implicated in human disease.

Infections caused by members of the *Mycobacterium chelonae-abscessus* complex remain a serious public health problem, and their role has expanded, with growing numbers of therapeutic interventions that disrupt the competency of the human immune system. Before 2001, only 2 species, *M. chelonae* and *M. abscessus,* were recognized as members of the complex. Since that time, 4 new species have been added to the complex: *M. immunogenum*, *M. massiliense*, *M. bolletii,* and *M. salmoniphilum*. With the exception of *M. salmoniphilum*, all members of the complex have been implicated in human disease (*1**–**4*). Recently investigators have noted a lack of separation of *M. bolletii* and *M. massiliense* from *M. abscessus* and have proposed they be classified as a subspecies of *M. abscessus* (*5*).

Members of the *M. chelonae-abscessus* complex represent *Mycobacterium* species that cause invasive skin and soft tissue infections, pneumonia, bloodstream infections, and abscesses in immunocompetent and immunocompromised hosts (*6**,**7*). Definitive identification by the clinical laboratory is needed for outbreak detection and for performance of susceptibility testing for patient management. Currently, the taxonomic relationships among members of the *M. chelonae-abscessus* complex lack clarity. The species are biochemically inert and their genetic signatures by partial 16S rRNA gene sequencing are often similar, which makes identification a great challenge for clinical laboratories.

In 2007, we detected a group of clinical isolates that were misidentified as *M. chelonae* with an atypical antimicrobial drug susceptibility profile. All isolates were from Pennsylvania, and as an interim identification, we labeled these isolates as CV for *M. chelonae* variant. Our discovery prompted a large multistate investigation that involved obtaining clinical correlation, retrospective and prospective collections of isolates with similar CV characteristics, and examination of a large set of known clinical isolates and type strains from the *M. chelonae-abscessus* complex. Given the taxonomic complexities and current ambiguities within the *M. chelonae-abscessus* group, we performed a comprehensive analysis of the potentially new species, including DNA-DNA hybridization, multilocus sequencing, and deep multilocus sequencing. We describe a new pathogen, *M. franklinii* sp. nov., a proposed new member of the *M. chelonae-abscessus* complex that was isolated from 26 patients in the United States. We discuss its potential role in human disease.

## Methods

### Isolates

All available type strains of *M. chelonae-abscessus* complex were obtained from American Type Culture Collection (ATCC), Collection of Institut Pasteur, or Culture Collection, University of Göteborg, Sweden. Previously identified clinical isolates of *M. abscessus*, and *M. chelonae* by partial 16S rRNA gene sequencing and internal transcribed spacer (ITS) PCR were retrieved for comparative analysis. A subset of these isolates was described in a prior study (*8*). CVs were defined as isolates that were cefoxitin susceptible or showed intermediate resistance, and identified as *M. chelonae* by partial 16S rRNA gene sequencing and ITS PCR (*9*) by Associated Regional and University Pathologists Laboratories and the Hospital of the University of Pennsylvania or by failure to amplify *hsp65* by PCR restriction fragment length polymorphism analysis (*10**,**11*) by the University of Texas Health Science Center. Microbiologic and medical records were reviewed for clinical information for select isolates. Clinical case reviews were conducted under institutional review board approved protocols at Associated Regional and University Pathologists Laboratories, Children’s Hospital of Philadelphia, University of Pennsylvania, and the University of Texas Health Science Center. Study isolates CV002 (ATCC [pending] and DSMZ 45524) and CV005 (ATCC [pending]) have been deposited into culture collections.

### Multilocus Sequencing

DNA extractions, PCR, and sequencing reactions were performed as described (*8*). Amplifications and sequencing reactions were performed by using primers specific for ≈1,400 bp of the 16S rRNA 5F (5′-TTGGAGAGTTTGATCCTGGCTC-3′) and 1492R (5′-ACGGITACCTTGTTACGACTT-3′), ≈700 bp of *rpoB,* ≈400 bp of *sodA* (*12*), ≈240 bp of the ITS (*13*), and ≈400 bp of *hsp65* (*11*) genes or region. Sequence alignments and phylogenetic trees were constructed by using neighbor-joining method with Kimura 2-parameter distance correction model and 1,000 bootstrap replications in MEGA4 (*14*). Only unique sequences (sequevars) were included in the trees.

### Standard for Identification

Final species identifications were based on comparisons of sequences for the full 16S rRNA and the partial *rpoB* genes to GenBank references of type strains. Full-length 16S rRNA sequences were used in this study, and we used 99.5% shared identity for identification to a type strain sequence. Species identification for *rpoB* gene was based on an identity of 98.0%–100% as outlined by Adekambi et al. (*12**,**15*).

### DNA-DNA Hybridization

Purified DNA of the type strains and the patient isolates CV002, CV004, CV005, CV006, and CV005 was prepared as described (*16*). CV002 and CV005 strains were labeled with [^32^P] dCTP using the Nick Translation Kit (Invitrogen, Carlsbad, CA, USA). Labeled DNA from the CV002 was hybridized with unlabeled DNA from isolates CV002, CV004, CV005, CV006, and CV015 and with unlabeled DNA from the type strains. Labeled DNA from patient isolate CV005 was then hybridized with unlabeled DNA from isolates CV002 and CV005. The reciprocal experiment was performed because of the nearness of CV005 to the 70% cut-off designated by Wayne (*17*) and the 0% divergence obtained in the first experiment. Hybridization was performed as previously described (*18*). All reactions were performed in duplicate at 70°C. The relative binding ratio (RBR) was calculated by using the method of Brenner et al. (*19*). The percentage divergence (calculated to the nearest 0.5%) was determined by assuming that each degree of heteroduplex instability, when compared with the melting temperature of the homologous duplex, was caused by 1% unpaired bases (*19*).

### Deep Multilocus Sequencing

Single-end DNA libraries of *M. bolletii* CIP 108541^T^, *M. chelonae* ATCC 35752^T^, *M. immunogenum* CIP 106684^T^, *M. massiliense* CCUG 48898^T^, and CV002 were prepared using Illumina DNA Sample Kit (Illumina, Inc., San Diego, CA, USA) according to manufacturer’s recommendations. Sequencing was performed in individual flow cell lanes on the Illumina Genome Analyzer (Illumina, Inc.) at the University of Utah Huntsman Cancer Institute Core Sequencing Facility.

De novo assembly of raw Illumina sequence data was achieved by using Velvet software (*20*). Velvet was run in 2 parts, velveth and velvetg. For velveth, the hash length was set at 23 (value was selected by calculations in the software manual); default settings were used for all other parameters. In velvetg, the –cov cutoff value was set to auto (setting allows software to automate appropriate coverage cutoff), and –min_contig_lgth was set to 100; all other settings were default.

The genome of *M. abscessus* CIP 104536^T^ (*21*) was used as the source reference set of 123 genes that were identified as likely core genome components for the phylum *Actinobacter* by Ventura et al. (*22*). The set of reference genes was randomly divided into 5 similarly sized sets to facilitate analysis. SeqMan (DNASTAR Inc., Madison, WI, USA) was used to align the assembled contigs from the sequenced species against each of the sets of reference genes. DNA and inferred amino acid sequences were aligned using MEGA. Only near full-length genes were used in future comparisons. Confirming the translation of the gene was in the correct reading frame relative to the different isolates substantiated quality of each assembled gene. The DNA and amino acid sequences were concatenated for each isolate and sequences alignments and phylogenetic trees were constructed in MEGA using the neighbor-joining method. Kimura 2-parameter distance correction was used for DNA and Poisson correction model was used for amino acid trees; 1,000 bootstrap replications were used for each tree constructed.

### Susceptibility Testing

We determined antimicrobial susceptibility by broth microdilution using the recommended Clinical and Laboratory Standards Institute guidelines for rapidly growing *Mycobacterium* spp (*23*). Some isolates were not tested for all antimicrobial agents and the concentrations of antimicrobial agents that were tested varied in the panels. MICs of clarithromycin were assessed at 3 days.

## Results

### Identification of Isolates by Multilocus Sequencing

We obtained 6 type strains representing all members of the *M. chelonae-abscessus* complex. All type strains and all 127 archived isolates underwent multilocus sequencing. For the 127 archived clinical isolates, we identified 64 *M. abscessus*, 58 *M.* *chelonae*, and 5 *M. massiliense* isolates. We designated an additional 26 isolates as CV on the basis of our case definition. All 26 CV isolates underwent *rpoB* gene sequencing, and a subset (n = 11) underwent multilocus sequencing. Unique sequevars are designated among the species *M. abscessus, M. massiliense, M. bolletii, M. chelonae,* and CV isolates.

#### 16S rRNA Gene

Alignments of 1,341 bp of the 16S rRNA gene show 8 unique sequevars from 14 variable bp positions (Figure 1). *M. chelonae* isolates had the most variability with 4 sequevars and an intraspecies variability of 0.2% (1–3 bp). No *M. chelonae* clinical isolates shared 100% identity to the type strain ATCC 35758^T^. *M. abscessus* isolates had 2 sequevars differing by 1 bp. Two *M. massiliense* sequevars were observed and differed by 1 bp with 1 sequevar identical to an *M. abscessus* sequevar. A single sequevar was found for the CV isolates, and was identical to the type strain of *M. chelonae*.

**Figure 1:** Neighbor-joining tree of a 1,341-bp region of unique 16S rRNA gene sequences of 138 clinical isolates and reference strains of the *Mycobacterium chelonae-abscessus* complex. Branch support is recorded at nodes as a percentage of 1,000 bootstrap iterations. Clinical isolates are labeled by the identification, followed by the sequevar group and the number of isolates. Scale bar indicates nucleotide substitutions per site. CIP, Collection of Institute Pasteur; CCUG, Culture Collection, University of Göteborg, Sweden; CV, *M. chelonae* variant; ATCC, American Type Culture Collection.

#### ITS Region

Alignments of 233 bp of the ITS region show 18 unique sequevars from 30 variable positions (Figure 2). *M. chelonae* isolates had the most variability with 12 sequevars and an intraspecies variability of 1.7% (1–4 bp). No *M. chelonae* clinical isolates shared 100% identity to the type strain ATCC 35758^T^. *M. abscessus* isolates showed 4 sequevars with an intraspecies variability of 0.9% (1–2 bp). One *M. massiliense* sequevar was observed, which was identical to 1 of the *M. abscessus* sequevars. Two sequevars were found for the CV isolates and differed by 2 bp (0.9%). The CV sequevars were most closely associated with *M. salmoniphilum* ATCC 13758^T^ at 98.7% (3 bp).

**Figure 2:** Neighbor-joining tree of 233 bp of unique internal transcribed spacer region sequences of 138 clinical isolates and reference strains of the *Mycobacterium chelonae-abscessus* complex. Branch support is recorded at nodes as a percentage of 1,000 bootstrap iterations. Clinical isolates are labeled by the identification, followed by the sequevar group and the number of isolates. Scale bar indicates nucleotide substitutions per site. ATCC, American Type Culture Collection; CV, *M. chelonae* variant; CIP, Collection of Institute Pasteur; CCUG, Culture Collection, University of Göteborg, Sweden.

#### *hsp65* Gene

Alignments of 361 bp of the *hsp65* gene show 11 unique sequevars from 41 variable positions (Figure 3). *M. chelonae* isolates had the most variability with 5 sequevars and an intraspecies variability of 1.9% (1–7 bp). No *M. chelonae* clinical isolates shared 100% identity to the type strain ATCC 35758^T^. *M. abscessus* isolates had 3 sequevars with an intraspecies variability of 1.7% (1–6 bp). One *M. massiliense* sequevar was observed and identical to 1 of the *M. abscessus* sequevars. Two sequevars were found for the CV isolates and differed by 1 bp (0.3%). The CV sequevars were most closely associated with *M. immunogenum* CIP 106684^T^ at 98.6% (5 bp).

**Figure 3:** Neighbor-joining tree of a 361-bp region of unique heat-shock protein 65 gene sequences of 138 clinical isolates and reference strains of the *Mycobacterium chelonae-abscessus* complex. Branch support is recorded at nodes as a percentage of 1,000 bootstrap iterations. Clinical isolates are labeled by the identification, followed by the sequevar group and the number of isolates. Scale bar indicates nucleotide substitutions per site. ATCC, American Type Culture Collection; CIP, Collection of Institute Pasteur; CV, *M. chelonae* variant; CCUG, Culture Collection, University of Göteborg, Sweden.

#### *sodA* Gene

Alignments of 426 bp of the *sodA* gene show 15 unique sequevars from 66 variable positions (Figure 4). *M. chelonae* isolates had the most variability with 9 sequevars and an intraspecies variability of 1.9% (1–8 bp). No *M. chelonae* clinical isolates shared 100% identity to the type strain ATCC 35758^T^. *M. abscessus* isolates had 3 sequevars with an intraspecies variability of 0.7% (1–3 bp). One *M. massiliense* sequevar was observed, which was identical to 1 of the *M. abscessus* sequevars. Two sequevars, which differed by 11 bp (2.6%), were found for the CV isolates. The CV sequevars were most closely associated with *M. immunogenum* CIP 106684^T^ at 95.8% (18 bp).

**Figure 4:** Neighbor-joining tree of a 426-bp region of unique *sodA* gene sequences of 138 clinical isolates and reference strains of the *Mycobacterium chelonae-abscessus* complex. Branch support is recorded at nodes as a percentage of 1,000 bootstrap iterations. Clinical isolates are labeled by the identification, followed by the sequevar group and the number of isolates. Scale bar indicates nucleotide substitutions per site. ATCC, American Type Culture Collection; CIP, Collection of Institute Pasteur; CV, *M. chelonae* variant; CCUG, Culture Collection, University of Göteborg, Sweden.

#### *rpoB* Gene

Alignments of 676 bp of region of the *rpoB* gene show 19 unique sequevars from 73 variable positions (Figure 5). *M. chelonae* isolates had the most variability with 9 sequevars and an intraspecies variability of 1.5% (1–10 bp). One *M. chelonae* clinical isolate shared 100% identity to the type strain ATCC 35758^T^. *M. abscessus* isolates had 4 sequevars with an intraspecies variability of 0.7% (1–5 bp). The largest sequevar of *M. abscessus* clinical isolates had 100% identity to the type strain of *M. abscessus*. Two *M. massiliense* sequevars were observed and differed by 2 bp (0.3%). Neither sequevar shared 100% identity with the type strain for *M. massiliense*. Four sequevars were observed among the CV isolates. They had an interspecies variability of 1.8% (2–11 bp). The CV sequevars were most closely associated with *M. chelonae* CIP 106684^T^ at 95.1% (33 bp). Sequevar results are summarized in Table 1 in the Technical Appendix.

**Figure 5:** Neighbor-joining tree of a 676-bp region of unique *rpoB* gene sequences of 153 clinical isolates and reference strains of the *Mycobacterium chelonae-abscessus* complex. Branch support is recorded at nodes as a percentage of 1,000 bootstrap iterations. Clinical isolates are labeled by the identification, followed by the sequevar group and the number of isolates. Scale bar indicates nucleotide substitutions per site. ATCC, American Type Culture Collection; CV, *M. chelonae* variant; CIP, Collection of Institute Pasteur; CCUG, Culture Collection, University of Göteborg, Sweden.

### Deep Multilocus Sequencing

Full-length genes were successfully assembled for 43 genes from 5 type strains and 1 representative clinical CV isolate. Gene names and corresponding GenBank accession numbers are provided in the supplementary tables (Technical Appendix Table 2). Pair-wise alignments of the DNA and amino acid sequences were performed for each isolate (Technical Appendix Tables 3, 4). The 43 concatenated genes ranged from 41,580 to 41,619 bp and created an alignment of 41,792 nt including gaps. Comparisons of DNA percent identity ranged from 89.1% (*M. chelonae* to *M. abscessus*) to 98.3% (*M. abscessus* to *M. bolletii*). *M. massiliense*, *M. abscessus*, and *M. bolletii* showed a close association with percent identity range of 98.2%–98.3%. The novel CV isolate shared no greater than 90.5% identity with any type strain. Phylogenetic analysis shows closest relationship between *M. abscessus, M. massiliense*, and *M. bolletii* (Figure 6, panel A). The CV isolate is on a separate branch with nearly identical distances from *M. immunogenum* and *M. chelonae*.

**Figure 6:** Neighbor-joining tree of DNA (A) and amino acid (B) concatenated gene sequences of *Mycobacterium chelonae* variant (CV) isolates and reference strains of the *M. chelonae-abscessus* complex. Branch support is recorded at nodes as a percentage of 1,000 bootstrap iterations. Upper scale bar indicates nucleotide substitutions per site and lower scale bar indicates amino acid substitutions per site. CIP, Collection of Institute Pasteur; CCUG, Culture Collection, University of Göteborg, Sweden; CV, *M. chelonae* variant; ATCC, American Type Culture Collection.

Percent identity of amino acid comparisons ranged from 95.7% (*M. chelonae* to *M. abscessus*) to 99.6% (*M. abscessus* to *M. bolletii*). *M. massiliense*, *M. abscessus*, and *M. bolletii* showed a close association with percent identity range of 99.5%–99.6%. The novel CV isolate shared no greater than 96.1% identity with any type strain. Phylogenetic analysis shows closest relationship between *M. abscessus, M. massiliense*, and *M. bolletii* (Figure 6, panel B).

### DNA-DNA Hybridization

DNA-DNA hybridization studies of clinical isolates CV004, CV005, CV006, and CV015 with labeled patient isolate CV002 showed RBRs of 66 to 96% and %D values of 0.0 to 2.0%. The reciprocal DNA-DNA hybridization of patient isolate CV002 with labeled isolate CV005 showed an RBR of 100% and a %D of 0.5%. The DNA-DNA hybridization studies of the phylogenetically (16S rRNA gene) related type strains *M. abscessus*, *M. bolletii*, *M. chelonae*, *M. immunogenum*, *M. massiliense*, and *M. salmoniphilum* performed with the patient isolate CV002 showed RBRs of 15%–69% and %D values of 5.0%–8.0%. DNA-DNA hybridization results are summarized in Technical Appendix Table 5.

### Susceptibility Testing

Susceptibilities by broth microdilution were available for most *M. chelonae, M. abscessus, M. massiliense,* and CV isolates (Table A1). The MICs for the first 3 taxa were comparable those in to previous reports (*24*). With the exception of 1 isolate, all isolates of *M. chelonae* were cefoxitin resistant (MIC >128 µg/mL), and *M. abscessus* and *M. massiliense* were cefoxitin intermediate (MIC 32–64 µg/mL). All 3 taxa were minocycline resistant. Unlike *M. chelonae*, CV isolates were intermediate (88%) or susceptible (12%) to cefoxitin.

### Clinical Spectrum and Characteristics of CV Isolates

The most common source for the CV isolates was respiratory (n = 20) (Technical AppendixTable 6). The remaining 6 CV isolates had clinical sources that included skin (n = 2), granulomatous liver lesion (n = 1), central line infections (n = 2), and an unspecified body fluid. Most isolates (n = 15) were recovered from patients seen in 4 different hospitals or clinics in Pennsylvania. Eight patients with CV infection were seen at the Hospital of the University of Pennsylvania and 7 of 8 patients were adult females (ages 41–74 years) who acquired the mycobacterial infection as outpatients. Six charts were available for review, and partial information was available for 2 additional patients (patients CV007, CV008, CV010, CV012–CV014, CV034, and CV036).

The medical histories of the 6 patients with complete information fell into 2 groups: those with chronic sinusitis (2 patients) and those with lower respiratory symptoms (4 patients). All patients with lower respiratory symptoms and cultures positive for CV had underlying pulmonary disease (cystic fibrosis, primary ciliary dyskinesia, lung cancer, chronic obstructive pulmonary disease, recurrent pneumonia/bronchiectasis). No patients with lower respiratory symptoms received specific antibiotic therapy aimed at treating rapidly growing mycobacterial infection, although 1 patient received long term antimicrobial drug therapy for concomitant *M. avium* infection. Two patients with sinusitis were not treated with antimicrobial drugs, but both had sinus surgery with symptomatic improvement. One patient had 3 positive sputum cultures for a rapidly growing *Mycobacterium* over a 3-year period, 2 of which were shown by genetic sequencing to be CV organisms (CV014 was selected for further study). No other patient had >1 positive culture for CV, if follow-up cultures were performed. Three patients had sequential or concomitant infections with *M. abscessus,* *M. avium-intracellulare,* or both bacteria.

The first isolate identified as a CV was discovered in 2005 from a patient in New York. The next isolate was not identified until 2007 from Pennsylvania. Overall, 23 (88%) of 26 were isolated in patients in the northeastern United States, and the remaining 3 isolates were recovered from patients in Minnesota, Oregon, and Colorado.

## Discussion

We describe the discovery of a new human pathogen with clinical features similar to *M. abscessus* that is implicated as a cause of infection for patients with chronic lung diseases, intravascular catheters, and chronic sinusitis. On the basis of our investigations, we propose CV isolates become a new member of the *M. chelonae-abscessus* complex and be named *Mycobacterium franklinii* sp. nov. *M. franklinii* (frank li′ ni I, N.L. masc. gen. n. *franklinii* of Franklin, pertaining to Benjamin Franklin, statesman, founder of the University of Pennsylvania, inventor, and scientist who helped create the nation’s first public hospital in Philadelphia, Pennsylvania, USA, the origin of the isolates).

The microorganisms are acid-fast, gram-positive bacilli, and colony morphology alone is not sufficient for differentiation from other rapidly growing *Mycobacterium* spp. Colonies are nonpigmented appearing on 5% sheep blood agar, Middlebrook 7H10 agar and egg-based Lowenstein-Jensen slants in 2–5 days at temperatures between 24 and 37°C (optimally at 30°C). Even with molecular techniques, underrecognition of this new pathogen is not surprising because it shares 100% full 16S rRNA gene identity with *M.* *chelonae* and has sequence variation in the *hsp65* gene that results in inconsistent amplification with typical diagnostic primers for sequence or PCR restriction fragment length polymorphism analysis (*10**,**11*). However, diagnosis of *M. franklinii* infection is essential because it is more susceptible to antimicrobial drugs than other members of the *M. chelonae-abscessus* complex, and its susceptibility pattern with cefoxitin was a distinguishing characteristic leading to its discovery.

Multilocus sequencing on a population of *M. chelonae-abscessus* complex isolates using 5 DNA regions enabled us to examine a population of closely related isolates to accurately assess species variability. *M. franklinii* shared complete 16S rRNA gene sequence identity with the type strain of *M. chelonae*, but was differentiated from *M. chelonae* and other members of the *M. chelonae-abscessus* complex by partial sequencing of *rpoB, hsp65, sodA,* and ITS DNA targets. Concatenated analysis of 43 genes (≈40,000 bp) from deeper sequencing of *M. franklinii* demonstrated that this novel species shares <90.5% identity with any other *M. chelonae-abscessus* group member. DNA-DNA hybridization analysis also supports the novel classification with its low relative binding ratios and higher percent divergence from all other *M. chelonae-abscessus* complex type strains. Cefoxitin susceptibility or intermediate susceptibility is another distinguishing feature. Preliminary testing on 6 *M. franklinii* isolates revealed inducible resistance (data not shown) in 50% of the isolates upon prolonged clarithromycin incubation (14 days). This finding suggests that similar to *M. abscessus*, isolates of this species may have an inducible *erm* gene.

The pathophysiology of diseases associated with *M. franklinii* is largely unknown. Most *M. franklinii* isolates were from respiratory sources and from patients with underlying lung conditions. Three of these disorders (cystic fibrosis, primary ciliary dyskinesia, and recurrent pneumonia) had associated bronchiectasis, which is a known risk factor for *M. abscessus* and *M. massiliense* nodular lung disease but not for *M. chelonae* (*6**,**25*). It is unclear whether this microorganism causes respiratory tract disease, or simply colonizes damaged airways and sinuses. Similar to patients with *M. abscessus* and *M.* *chelonae* infections, we found 2 cases each of sinusitis and catheter-associated infection from *M. franklinii* (*26*). The association with chronic sinusitis presumably relates to sinus washes using tap water rinses in previously diseased sinuses. Although the exact reservoir of *M. franklinii* is unknown, a recent study in the Netherlands by Van Ingen et al. reported 2 isolates from tap and shower water based on *rpoB* gene sequence (*27*), and these 2 isolates shared 99.6% identity to our *M. franklinii* sequevar A *rpoB* gene sequence. The observation in the Netherlands suggests an environmental source for this organism, and it is likely that the novel species is regionally specific and can survive in municipal water sources. This hypothesis would be supported by the large number of cases in a focused region in Pennsylvania.

Our population analyses of clinical isolates of *M. chelonae* demonstrate a lack of taxonomic clarity. Additionally, our investigations lend further evidence that species distinctions for *M.* *bolletii* and *M. massiliense* may be inappropriate and support the recent proposal to modify their classifications (*5**,**28*). Taxonomic uncertainty likely arises as our understanding of microbial phylogeny expands with rapid advances in technologies and often results in inconsistent standards being applied for species designations. For example, DNA-DNA hybridization is a relatively standard technique, but upon review of 14 species descriptions in 2009 only 5 were supported by using DNA-DNA hybridization (*29**–**36*).

The discovery of an emerging pathogen should be taken in the context of microbial ecology and evolution, the interaction between host and microbe, and factors of virulence. This investigation underscores the need for accurate identification of *Mycobacterium* spp. for detection of a new pathogen. The interplay between colonization and disease is not clearly defined, but we demonstrate its role in central line infections and for patients with sinopulmonary disease. *M. franklinii* may have newly emerged as a human pathogen over the past 5 years, or it has been involved in human disease previously and was unrecognized. In order to further our understanding of this pathogen and its role in disease, greater surveillance and awareness is necessary. At this time, clinical laboratories can identify *M. franklinii* by sequencing based assays that target either the ITS region (between 16S and 23S rRNA genes), *hsp65*, *rpoB*, and *sodA* genes, or by complete 16S rRNA gene sequence analysis in conjunction with cefoxitin and minocycline susceptibility patterns. The type strain, CV002 (ATCC [pending] and DSMZ 45524), was isolated from a skin lesion.

## Appendix

**Table A1:** Antimicrobial drug susceptibility profiles of CV isolates compared with *Mycobacterium chelonae, M. massiliense,* and *M. abscessus* isolates*

Antimicrobial drug	*M. abscessus*, n = 41					*M. massiliense*, n = 5					*M. chelonae*, n = 58					CV, n = 25			
	Range	S, %	I, %	R, %		Range	S, %	I, %	R, %		Range	S, %	I , %	R, %		Range	S, %	I, %	R, %
Amikacin	8−32	73.2	26.8	0.0		8−32	60.0	40.0	0.0		8−>256	32.8	51.7	15.5		8−32	92.0	8.0	0.0
Cefoxitin	16−64	9.8	90.2	0.0		16−32	40.0	60.0	0.0		64−> 512	0.0	1.7	98.3		16−64	12.0	88.0	0.0
Ciprofloxacin†	<0.25−>32	2.4	0.0	97.6		4−16	0.0	0.0	100.0		1−>32	8.6	17.2	74.2		<0.25−8	60.0	20.0	20.0
Clarithromycin	<0.12−4	97.6	2.4	0.0		<0.12	100.0	0.0	0.0		<0.12−>64	98.3	0.0	2.4		<0.12−1	100.0	0.0	0.0
Gatifloxacin‡	<0.5−>16	2.4	14.6	83.0		4−16	0.0	20.0	80.0		1−>16	31.0	44.8	24.2		<0.5−4§	80.0	20.0	0.0
Imipenem	1−32	14.6	53.6	31.8		8	0.0	100.0	0.0		<0.5−64	10.3	34.5	55.2		1−64	16.0	44.0	40.0
Linezolid	4−32	43.9	36.6	19.5		8−16	20.0	60.0	20.0		<0.1−32	81.0	13.8	5.2		1−32	72.0	24.0	4.0
Minocycline	8−64	0.0	4.9	95.1		64	0.0	0.0	100.0		<0.5−>64	15.5	3.5	81.0		<0.5−16¶	90.9	9.1	0.0
Tobramycin	4−32	2.4	22.0	75.6		8−32	0.0	0.0	100.0		1−>128	98.3	0.0	1.7		4−16#	62.5	29.2	8.3
Trimethoprim/ sulfamethoxazole	4/76−>16/304	0.0	–	100.0		>16/304	0.0	–	100.0		<0.5/9.5−>16/304	5.2	–	94.8		1/9−>16/304	8.0	–	92.0

*CV, *M. chelonae* variant; S, susceptible; I, intermediate; R, resistant; –, not applicable. One CV isolate did not grow on solid media. †Read at 3 days ‡No CLSI breakpoint recommendation has been proposed for gatifloxacin. Manufacturer recommendation of 4 used as the intermediate breakpoint. §10 isolates tested. ¶22 isolates tested. #24 isolates tested.

## References

[1] Wallace RJ Jr, Zhang Y, Wilson RW, Mann L, Rossmoore H. Presence of a single genotype of the newly described species *Mycobacterium immunogenum* in industrial metalworking fluids associated with hypersensitivity pneumonitis. Appl Environ Microbiol. 2002;68:5580–4. 10.1128/AEM.68.11.5580-5584.200212406752PMC129929

[2] Adékambi T, Reynaud-Gaubert M, Greub G, Gevaudan MJ, La Scola B, Raoult D, Amoebal coculture of “*Mycobacterium massiliense*” sp. nov. from the sputum of a patient with hemoptoic pneumonia. J Clin Microbiol. 2004;42:5493–501. 10.1128/JCM.42.12.5493-5501.200415583272PMC535245

[3] Adékambi T, Berger P, Raoult D, Drancourt M. *rpoB* gene sequence-based characterization of emerging non-tuberculous mycobacteria with descriptions of *Mycobacterium bolletii* sp. nov., *Mycobacterium phocaicum* sp. nov. and *Mycobacterium aubagnense* sp. nov. Int J Syst Evol Microbiol. 2006;56:133–43. 10.1099/ijs.0.63969-016403878

[4] Whipps CM, Butler WR, Pourahmad F, Watral VG, Kent ML. Molecular systematics support the revival of *Mycobacterium salmoniphilum* (ex Ross 1960) sp. nov., nom. rev., a species closely related to *Mycobacterium chelonae.* Int J Syst Evol Microbiol. 2007;57:2525–31. 10.1099/ijs.0.64841-017978213

[5] Leao SC, Tortoli E, Euzéby JP, Garcia MJ. Proposal that the two species *Mycobacterium massiliense* and *Mycobacterium bolletii* be reclassified as *Mycobacterium abscessus* subsp. *bolletii* comb. nov., designation of *Mycobacterium abscessus* subsp. *abscessus* subsp. nov., and emendation of *Mycobacterium abscessus.* Int J Syst Evol Microbiol. 2010; [Epub ahead of print].10.1099/ijs.0.023770-021037035

[6] Griffith DE, Girard WM, Wallace RJ Jr. Clinical features of pulmonary disease caused by rapidly growing mycobacteria. An analysis of 154 patients. Am Rev Respir Dis. 1993;147:1271–8.848464210.1164/ajrccm/147.5.1271

[7] Wallace RJ Jr, Swenson JM, Silcox VA, Good RC, Tschen JA, Stone MS. Spectrum of disease due to rapidly growing mycobacteria. Rev Infect Dis. 1983;5:657–79. 10.1093/clinids/5.4.6576353528

[8] Simmon KE, Pounder JI, Greene JN, Walsh F, Anderson CM, Cohen S, Identification of an emerging pathogen, *Mycobacterium massiliense*, by *rpoB* sequencing of clinical isolates collected in the United States. J Clin Microbiol. 2007;45:1978–80. 10.1128/JCM.00563-0717409204PMC1933107

[9] Cloud JL, Hoggan K, Belousov E, Cohen S, Brown-Elliott BA, Mann L, Use of the MGB Eclipse system and SmartCycler PCR for differentiation of *Mycobacterium chelonae* and *M. abscessus.* J Clin Microbiol. 2005;43:4205–7. 10.1128/JCM.43.8.4205-4207.200516081979PMC1233948

[10] Steingrube VA, Gibson JL, Brown BA, Zhang Y, Wilson RW, Rajagopalan M, PCR amplification and restriction endonuclease analysis of a 65-kilodalton heat shock protein gene sequence for taxonomic separation of rapidly growing mycobacteria. J Clin Microbiol. 1995;33:149–53.769903210.1128/jcm.33.1.149-153.1995PMC227898

[11] Telenti A, Marchesi F, Balz M, Bally F, Bottger EC, Bodmer T. Rapid identification of mycobacteria to the species level by polymerase chain reaction and restriction enzyme analysis. J Clin Microbiol. 1993;31:175–8.838180510.1128/jcm.31.2.175-178.1993PMC262730

[12] Adékambi T, Colson P, Drancourt M. *rpoB*-based identification of nonpigmented and late-pigmenting rapidly growing mycobacteria. J Clin Microbiol. 2003;41:5699–708. 10.1128/JCM.41.12.5699-5708.200314662964PMC308974

[13] Roth A, Reischl U, Streubel A, Naumann L, Kroppenstedt RM, Habicht M, Novel diagnostic algorithm for identification of mycobacteria using genus-specific amplification of the 16S-23S rRNA gene spacer and restriction endonucleases. J Clin Microbiol. 2000;38:1094–104.1069900310.1128/jcm.38.3.1094-1104.2000PMC86348

[14] Tamura K, Dudley J, Nei M, Kumar S. MEGA4: Molecular Evolutionary Genetics Analysis (MEGA) software version 4.0. Mol Biol Evol. 2007;24:1596–9. 10.1093/molbev/msm09217488738

[15] Adékambi T, Drancourt M. Dissection of phylogenetic relationships among 19 rapidly growing *Mycobacterium* species by 16S rRNA, *hsp65, sodA, recA* and *rpoB* gene sequencing. Int J Syst Evol Microbiol. 2004;54:2095–105. 10.1099/ijs.0.63094-015545441

[16] Loeffelholz MJ, Scholl DR. Method for improved extraction of DNA from *Nocardia asteroides.* J Clin Microbiol. 1989;27:1880–1.267103610.1128/jcm.27.8.1880-1881.1989PMC267689

[17] Wayne LG. International Committee on Systematic Bacteriology: announcement of the report of the ad hoc Committee on Reconciliation of Approaches to Bacterial Systematics. Zentralbl Bakteriol Mikrobiol Hyg [A]. 1988;268:433–4.321331410.1016/s0176-6724(88)80120-2

[18] Brenner DJ, McWhorter AC, Knutson JK, Steigerwalt AG. *Escherichia vulneris*: a new species of *Enterobacteriaceae* associated with human wounds. J Clin Microbiol. 1982;15:1133–40.710784310.1128/jcm.15.6.1133-1140.1982PMC272265

[19] Brenner DJ, Hickman-Brenner FW, Lee JV, Steigerwalt AG, Fanning GR, Hollis DG, *Vibrio furnissii* (formerly aerogenic biogroup of *Vibrio fluvialis*), a new species isolated from human feces and the environment. J Clin Microbiol. 1983;18:816–24.663046410.1128/jcm.18.4.816-824.1983PMC270912

[20] Zerbino DR, Birney E. Velvet: algorithms for de novo short read assembly using de Bruijn graphs. Genome Res. 2008;18:821–9. 10.1101/gr.074492.10718349386PMC2336801

[21] Ripoll F, Pasek S, Schenowitz C, Dossat C, Barbe V, Rottman M, Non mycobacterial virulence genes in the genome of the emerging pathogen *Mycobacterium abscessus.* PLoS ONE. 2009;4:e5660. 10.1371/journal.pone.000566019543527PMC2694998

[22] Ventura M, Canchaya C, Tauch A, Chandra G, Fitzgerald GF, Chater KF, Genomics of Actinobacteria: tracing the evolutionary history of an ancient phylum. Microbiol Mol Biol Rev. 2007;71:495–548. 10.1128/MMBR.00005-0717804669PMC2168647

[23] Woods GL, Brown-Elliott BA, Desmond EP, Hall GS, Heifets L, Pfyffer GE, Susceptibility testing of mycobacteria, nocardiae, and other aerobic actinomycetes; approved standard. National Committee on Clinical and Laboratory Standards;23:M24-A. Wayne (PA): The Committee; 2003.31339680

[24] Swenson JM, Wallace RJ Jr, Silcox VA, Thornsberry C. Antimicrobial susceptibility of five subgroups of *Mycobacterium fortuitum* and *Mycobacterium chelonae.* Antimicrob Agents Chemother. 1985;28:807–11.408386310.1128/aac.28.6.807PMC180333

[25] Roux AL, Catherinot E, Ripoll F, Soismier N, Macheras E, Ravilly S, Multicenter study of prevalence of nontuberculous mycobacteria in patients with cystic fibrosis in France. J Clin Microbiol. 2009;47:4124–8. 10.1128/JCM.01257-0919846643PMC2786646

[26] Redelman-Sidi G, Sepkowitz KA. Rapidly growing mycobacteria infection in patients with cancer. Clin Infect Dis. 2010;51:422–34. 10.1086/65514020617902

[27] van Ingen J, Blaak H, de Beer J, de Roda Husman AM, van Soolingen D. Rapidly growing nontuberculous mycobacteria cultured from home tap and shower water. Appl Environ Microbiol. 2010;76:6017–9. 10.1128/AEM.00843-1020639378PMC2935072

[28] Leao SC, Tortoli E, Viana-Niero C, Ueki SY, Lima KV, Lopes ML, Characterization of mycobacteria from a major Brazilian outbreak suggests that revision of the taxonomic status of members of the *Mycobacterium chelonae*-*M. abscessus* group is needed. J Clin Microbiol. 2009;47:2691–8. 10.1128/JCM.00808-0919571015PMC2738059

[29] Ben Salah I, Cayrou C, Raoult D, Drancourt M. *Mycobacterium marseillense* sp. nov., *Mycobacterium timonense* sp. nov. and *Mycobacterium bouchedurhonense* sp. nov., members of the *Mycobacterium avium* complex. Int J Syst Evol Microbiol. 2009;59:2803–8. 10.1099/ijs.0.010637-019628609

[30] Hennessee CT, Seo JS, Alvarez AM, Li QX. Polycyclic aromatic hydrocarbon-degrading species isolated from Hawaiian soils: *Mycobacterium crocinum* sp. nov., *Mycobacterium pallens* sp. nov., *Mycobacterium rutilum* sp. nov., *Mycobacterium rufum* sp. nov. and *Mycobacterium aromaticivorans* sp. nov. Int J Syst Evol Microbiol. 2009;59:378–87. 10.1099/ijs.0.65827-019196782

[31] Okazaki M, Ohkusu K, Hata H, Ohnishi H, Sugahara K, Kawamura C, *Mycobacterium kyorinense* sp. nov., a novel, slow-growing species, related to *Mycobacterium celatum*, isolated from human clinical specimens. Int J Syst Evol Microbiol. 2009;59:1336–41. 10.1099/ijs.0.000760-019502312

[32] Tortoli E, Baruzzo S, Heijdra Y, Klenk HP, Lauria S, Mariottini A, *Mycobacterium insubricum* sp. nov. Int J Syst Evol Microbiol. 2009;59:1518–23. 10.1099/ijs.0.003459-019502346

[33] van Ingen J, Al-Hajoj SA, Boeree M, Al-Rabiah F, Enaimi M, de Zwaan R, *Mycobacterium riyadhense* sp. nov., a non-tuberculous species identified as *Mycobacterium tuberculosis* complex by a commercial line-probe assay. Int J Syst Evol Microbiol. 2009;59:1049–53. 10.1099/ijs.0.005629-019406791

[34] van Ingen J, Boeree MJ, de Lange WC, de Haas PE, van der Zanden AG, Mijs W, *Mycobacterium noviomagense* sp. nov.; clinical relevance evaluated in 17 patients. Int J Syst Evol Microbiol. 2009;59:845–9. 10.1099/ijs.0.001511-019329618

[35] van Ingen J, Boeree MJ, Kosters K, Wieland A, Tortoli E, Dekhuijzen PN, Proposal to elevate *Mycobacterium avium* complex ITS sequevar MAC-Q to *Mycobacterium vulneris* sp. nov. Int J Syst Evol Microbiol. 2009;59:2277–82. 10.1099/ijs.0.008854-019620376

[36] van Ingen J, Lindeboom JA, Hartwig NG, de Zwaan R, Tortoli E, Dekhuijzen PN, *Mycobacterium mantenii* sp. nov., a pathogenic, slowly growing, scotochromogenic species. Int J Syst Evol Microbiol. 2009;59:2782–7. 10.1099/ijs.0.010405-019625425

# Research article: *Mycobacterium chelonae-abscessus* Complex Associated with Sinopulmonary Disease, Northeastern USA

**DOI:** 10.3201/eid1709.101667

**Authors:** Keith E. Simmon, Barbara A. Brown-Elliott, Perry G. Ridge, Jacob D. Durtschi, Linda Bridge Mann, E. Susan Slechta, Arnold G. Steigerwalt, Benjamin D. Moser, Anne M. Whitney, June M. Brown, Karl V. Voelkerding, Karin L. McGowan, Anne F. Reilly, Thomas J. Kirn, W. Ray Butler, Paul H. Edelstein, Richard J. Wallace, Cathy A. Petti

**Affiliations:** Author affiliations: Associated Regional and University Pathologists Institute for Clinical and Experimental Pathology, Salt Lake City, Utah, USA (K.E. Simmon, P.G. Ridge, J.D. Durtschi, E.S. Slechta, K.V. Voelkerding, C.A. Petti);; University of Texas Health Science Center, Tyler, Texas, USA (B.A. Brown-Elliott, L.B. Mann, R.J. Wallace, Jr.);; Centers for Disease Control and Prevention, Atlanta, Georgia, USA (A.G. Steigerwalt, B.D. Moser, A.M. Whitney, J.M. Brown, W.R. Butler);; University of Utah School of Medicine, Salt Lake City, Utah, USA (K.V. Voelkerding, C.A. Petti);; University of Pennsylvania School of Medicine at Children’s Hospital of Philadelphia, Philadelphia, Pennsylvania, USA (K.F. McGowan, A.F. Reilly);; Robert Wood Johnson Medical School, New Brunswick, New Jersey, USA (T.J. Kirn);; University of Pennsylvania Medical Center, Philadelphia (P.H. Edelstein)

**Keywords:** *Suggested citation for this article*: Simmon KE, Brown-Elliott BA, Ridge PG, Durtschi JD, Mann LB, Slechta ES, et al. *Mycobacterium chelonae-abscessus* complex associated with sinopulmonary disease, northeastern USA. Emerg Infect Dis [serial on the Internet]. 2011 Sep [*date cited*]. http://dx.doi.org/10.3201/eid1709.101667

## Earning CME Credit

To obtain credit, you should first read the journal article. After reading the article, you should be able to answer the following, related, multiple-choice questions. To complete the questions (with a minimum 70% passing score) and earn continuing medical education (CME) credit, please go to www.medscape.org/journal/eid. Credit cannot be obtained for tests completed on paper, although you may use the worksheet below to keep a record of your answers. You must be a registered user on Medscape.org. If you are not registered on Medscape.org, please click on the New Users: Free Registration link on the left hand side of the website to register. Only one answer is correct for each question. Once you successfully answer all post-test questions you will be able to view and/or print your certificate. For questions regarding the content of this activity, contact the accredited provider, CME@medscape.net. For technical assistance, contact CME@webmd.net. American Medical Association’s Physician’s Recognition Award (AMA PRA) credits are accepted in the US as evidence of participation in CME activities. For further information on this award, please refer to http://www.ama-assn.org/ama/pub/category/2922.html. The AMA has determined that physicians not licensed in the US who participate in this CME activity are eligible for *AMA PRA Category 1 Credits*™. Through agreements that the AMA has made with agencies in some countries, AMA PRA credit may be acceptable as evidence of participation in CME activities. If you are not licensed in the US, please complete the questions online, print the certificate and present it to your national medical association for review.

## Article Title: *Mycobacterium chelonae-abscessus* Complex Associated with Sinopulmonary Disease, Northeastern USA

## CME Questions

**1. Which of the following statements regarding the members of the *Mycobacterium chelonae-abscessus* complex is most accurate?**A. There are 22 known members of the *M. chelonae-abscessus* complexB. These mycobacteria can promote skin infections, pneumonia, and abscessesC. Infections occur only among immunocompromised hostsD. There are clear taxonomical relationships between members of the *M. chelonae-abscessus* complex**2. "*Mycobacterium franklinii*" shared which of the following gene sequences with *M. chelonae* in the current study?**A. *rpoB*B. *sodA*C. *hsp65*D. 16S rRNA**3. What was the most common source of "*M. franklinii*" in the current study?**A. SkinB. Liver lesionC. Central lineD. Respiratory**4. Which of the following statements regarding the antimicrobial susceptibility of "*M. franklinii*" is most accurate?**A. "*M. franklinii*" has the same susceptibility profile as other members of the *M. chelonae-abscessus* complexB. "*M. franklinii* " is more likely to be susceptible to minocycline compared with other members of the *M. chelonae-abscessus* complexC. "*M. franklinii* " is more likely to be susceptible to cefoxitin compared with other members of the *M. chelonae-abscessus* complexD. "*M. franklinii* " is more likely to be resistant to cefoxitin compared with other members of the *M. chelonae-abscessus* complex

## Activity Evaluation

**Table:** 

1. The activity supported the learning objectives.				
Strongly Disagree				Strongly Agree
1	2	3	4	5
2. The material was organized clearly for learning to occur.				
Strongly Disagree				Strongly Agree
1	2	3	4	5
3. The content learned from this activity will impact my practice.				
Strongly Disagree				Strongly Agree
1	2	3	4	5
4. The activity was presented objectively and free of commercial bias.				
Strongly Disagree				Strongly Agree
1	2	3	4	5

## Supplementary Material

Technical Appendix Study isolates, gene designations, similarity tables, DNA comparison, and case history information pertaining to the article.
